# Concise Review: Paracrine Functions of Vascular Niche Cells in Regulating Hematopoietic Stem Cell Fate

**DOI:** 10.5966/sctm.2016-0254

**Published:** 2016-09-13

**Authors:** Joshua P. Sasine, Kelly T. Yeo, John P. Chute

**Affiliations:** ^1^ Division of Hematology/Oncology, Department of Medicine, David Geffen School of Medicine, University of California, Los Angeles, Los Angeles, California, USA; ^2^ Jonsson Comprehensive Cancer Center, University of California, Los Angeles, Los Angeles, California, USA; ^3^ Eli and Edythe Broad Center for Regenerative Medicine and Stem Cell Research, University of California, Los Angeles, Los Angeles, California, USA

**Keywords:** Hematopoietic stem cells, Endothelial cell, Bone marrow stromal cells, Self‐renewal, Microenvironment

## Abstract

The functions of endothelial cells (ECs) in regulating oxygen delivery, nutrient exchange, coagulation, and transit of inflammatory cells throughout the body are well‐‐established. ECs have also been shown to regulate the maintenance and regeneration of organ‐specific stem cells in mammals. In the hematopoietic system, hematopoietic stem cells (HSCs) are dependent on signals from the bone marrow (BM) vascular niche for their maintenance and regeneration after myelosuppressive injury. Recent studies have demonstrated the essential functions of BM ECs and perivascular stromal cells in regulating these processes. In the present study, we summarize the current understanding of the role of BM ECs and perivascular cells in regulating HSC maintenance and regeneration and highlight the contribution of newly discovered EC‐derived paracrine factors that regulate HSC fate. Stem Cells Translational Medicine *2017;6:482–489*

**Significance Statement**

Recent studies have shown that blood stem cells require signals from the bone marrow microenvironment or niche for their survival and regeneration. In the present study, the current understanding of the interactions between blood stem cells and niche cells is summarized and the potential for niche‐derived secreted factors as therapeutic agents for regenerative medicine is highlighted.

## Introduction

Vascular endothelial cells (ECs) constitute the intimal lining of arteries, capillaries, and veins within the human vasculature and serve critical roles in the delivery of oxygen and nutrients, in the regulation of coagulation, and as the gatekeepers to inflammatory cells for entry into tissues 1, 2, 3. During development, ECs are necessary for the formation of the hematopoietic system and the emergence of definitive hematopoietic stem cells (HSCs) 4, 5, 6, 7. In the past decade, discoveries from several laboratories have demonstrated a critical function for ECs in the adult HSC niche, such that ECs regulate HSC maintenance and regeneration after stress or injury 8, 9, 10, 11, 12, 13, 14, in concert with other microenvironmental cues. Exquisite microanatomical studies have confirmed that the most primitive HSCs reside in bone marrow (BM) vascular and perivascular niches 15, 16. BM ECs regulate HSC fate, in part, via the elaboration of paracrine factors 17, 18, 19. Commensurate with these observations, recent studies have suggested that ECs also regulate the self‐renewal and regeneration of stem cells in nonhematopoietic organs, including the liver, brain, lung, and skin 1, 20, 21, 22, 23, 24, 25, 26, 27, 28, 29. In the present study, we describe the hematopoietic‐specific activities of BM ECs and highlight the potential for EC‐derived paracrine mechanisms to be translated into regenerative therapies for patients.

## Endothelial Cells and Hematopoietic Development

Within the embryo, development of the hematopoietic system occurs in multiple locations at various stages, including the extraembryonic yolk sac, fetal liver, spleen, and, finally, adult BM. Evidence exists for two discrete anatomic origins of hematopoietic activity, one extraembryonic and one intraembryonic 30. During the primitive streak stage, groups of mesodermal cells in the yolk sac form the extraembryonic blood islands (mouse gestational age E7.5). The peripheral cells differentiate into EC precursors (angioblasts), and the inner cells become primitive blood cells 31. The two lineages are so closely related in time and space that this led to a hypothesis regarding a common precursor, the “hemangioblast.” Blast colony tracing studies later provided direct evidence for the existence of the hemangioblast 5.

Before 1975, it was widely accepted that definitive HSCs originated from the yolk sac blood islands. To evaluate this dogma, Françoise Dieterlen‐Lièvre took advantage of the ability to distinguish chick and quail cell nuclei and grafted 2‐day‐old quail embryos on to chick yolk sacs of comparable developmental stages, before or shortly after the establishment of vascularization 32. The chick cells never gave rise to adult hematopoiesis; thus, although it contributes to embryonic hematopoiesis, the source of definitive long‐term HSCs was concluded to be intraembryonic.

The first definitive HSC known to be able to fully reconstitute the hematopoietic system on transplantation was identified in the aorto‐gonad‐mesonephros region (AGM) in mice and humans 5, 33, 34, 35. Yet other studies have suggested that yolk sac cells can mature into definitive HSCs provided they are transplanted into a newborn and not an adult mouse 36. In addition, a significant reservoir of HSCs can be found residing in the placenta during development 37, 38. HSCs from the AGM are presumed to colonize the fetal liver, where they give rise to definitive hematopoietic precursors. Several seminal studies using lineage tracing in both mice and zebrafish have shown that definitive HSCs arise from hemogenic endothelium within the ventral aspect of the dorsal aorta at E10.5 39, 40, 41. Runx1 is required for this process to occur 42, and mice lacking Flk1, a tyrosine kinase expressed on endothelial progenitor cells, fail to develop both vascular endothelium and blood islands during embryogenesis 43.

Later in development, HSCs reside in the fetal liver. Many questions regarding the fetal liver vascular niche remain, especially regarding the process of migration of HSCs from the fetal liver to the BM following birth. A recent study showed that nestin‐positive NG2^+^ pericytes associate with portal vessels, forming a fetal perivascular niche that promotes HSC expansion 7. A rapid loss of HSCs in the postnatal liver is associated with dramatic postnatal changes in portal vessel circulation after closure of the umbilical inlet at birth. A corresponding loss of niche cells, including nestin‐positive NG2^+^ pericytes, also occurs, because the portal vessels undergo a transition from neuropilin‐1^+^ephrin‐B2^+^ arteries to an EphB4^+^ vein phenotype. This suggests that the changes in postnatal circulation and hemodynamics alter the liver vasculature and are associated with the loss of HSC‐supporting cells. The HSCs then migrate to the BM wherein long‐term hematopoiesis persists postnatally.

## Conceptual History of the HSC Niche

In 1968, Wolf and Trentin directly implanted BM stroma into the spleens of irradiated recipients and showed that the proportions of outgrowing erythroid and myeloid colonies could be biased by their local environment, providing some evidence for microenvironmental influence of immature hematopoietic cell fate 44. The existence of a niche for stem cells within the BM was formally proposed by Schofield in 1978 45. At that time, Becker et al. showed that HSCs likely existed based on their research using the colony‐forming unit‐spleen (CFU‐S) assay 46. Schofield noted that the putative CFU‐S stem cells were less robust than cells of the BM at reconstituting hematopoiesis in irradiated animals and proposed the existence of a specialized location within the BM that maintained HSCs 45. However, definitive evidence of an HSC population and an HSC niche was lacking, and many questions regarding the factors that governed HSC differentiation and maintenance remained unanswered for decades. Some groups proposed stochastic mechanisms and others favored an “inductive” hypothesis in which the niche governed HSC fate. Over the subsequent decades, numerous studies clarified the critical role of BM microenvironment cells in regulating HSC fate.

## Anatomy of the Adult HSC Niche

In addition to HSCs and their progeny, the BM is composed of a diverse array of cells with specialized functions. These include vascular ECs, perivascular cells, osteoblasts, sympathetic nerves, adipocytes, macrophages, and many subsets of stromal cells 1, 47, 48. BM niche cells provide both positive and negative regulatory signals for HSCs. Adipocytes have been shown to negatively regulate HSC self‐renewal in vivo 49. Adipogenic differentiation of stromal cells also leads to increased adipocyte numbers in the BM, which, in turn, hampers hematopoietic recovery after injury 50. Bone‐degrading osteoclasts are dispensable for HSC maintenance in *op/op*, *Fos*‐deficient, and *Rankl*‐deficient mice, which lack osteoclasts 51, 52.

Most mammalian hematopoiesis occurs in the axial skeleton (so‐called red marrow) in the flat bones, such as the pelvis, sternum, skull, ribs, vertebrae, and the metaphyseal and epiphyseal ends of long bones. Other BM, composed of higher fat content (“yellow marrow”), can be found in the hollow interior of the diaphyseal portion (shaft) of the long bones. Arteries enter through the bone cortex, terminate in the endosteum (the connective tissue lining the inner surface of compact bone), and branch in the metaphysis or diaphysis of long bones 53, 54. Most of the branching arteries lie in the metaphysis, and the central diaphysis contains few, largely unbranched arteries 53. Some distal arterioles terminate at capillaries in the endosteum, although most termination points are found in the metaphysis 53. Near the bone, the arterioles open up and anastomose with a plexus of venous sinuses 53. These venous sinuses drain via collecting venules that lead back centrally to the central longitudinal vein 53. The arterioles associate more closely with the endosteal region and are ensheathed exclusively by rare NG2^+^ (also known as CSPG4^+^), nestin^bright^ pericytes, which are distinct from sinusoid‐associated leptin receptor (LepR)^+^ or Nestin^dim^ perivascular cells 16. Veins are located in the central diaphysis, where they connect to metaphyseal capillaries. The venous sinuses are thin‐walled, consisting of a layer of flat ECs with little to no basement membrane and covered with pericytes and perivascular stromal cells, including C‐X‐C motif chemokine ligand 12 (CXCL12) abundant reticular cells (CAR cells) 55 and Lepr^+^ cells 16, 56, 57. The BM does not have lymphatic drainage 58, and all vessels are interspersed within a meshwork of trabecular bone. This anatomy establishes a circular pattern to the blood flow, from the center of the marrow cavity toward the periphery and back again.

Defining the anatomic location of HSCs in the adult BM as it relates to distinct niche cells has proved to be challenging owing to the difficulty in retaining histological integrity when sectioning bone, the number of markers necessary to identify HSCs, the limitations of microscopy, and the close proximity of bone and vascular elements throughout the BM. The discovery of a pattern of SLAM family receptor expression (CD150^+^CD244^−^CD48^−^) on HSCs catalyzed the ability of researchers to localize HSCs in the niche. Most SLAM^+^ HSCs were found in that study to be associated with sinusoidal endothelium 59. Previously, up to 10 markers had been necessary to identify HSCs, and even then, the purity of the population as long‐term HSCs rarely exceeded 20% 60.

It has also been shown that the local oxygen concentration and HSC metabolic state in the BM contributes to regional differences in HSC spatial localization 16, 61, 62, 63, 64, 65, 66. Quiescent HSCs have been shown to have lower levels of intracellular reactive oxygen species (ROS), and high ROS levels damage HSC self‐renewal capacity and promote HSC exhaustion 61, 62, 63, 64, 65. Quiescent HSCs with lower levels of ROS have been shown to reside adjacent to arteriolar blood vessels rather than sinusoids 16, 66, and conditional depletion of the ensheathing arteriolar pericytes induces HSC cycling, with ensuing reduced functional long‐term repopulating ability 16. However, if the metabolic state is ignored, this differential vascular localization no longer applies, and HSCs are found throughout arteriolar and sinusoidal niches 66. Compared with arterioles, the more permeable BM sinusoids promote HSC activation. Furthermore, HSC exposure to blood plasma was shown to increase HSC ROS levels, augmenting their migration and differentiation and compromising their long‐term repopulation abilities and survival 66. In contrast, Acar et al. demonstrated that HSCs were localized primarily in the central BM away from bone surfaces, in the diaphysis relative to the metaphysis, and close to sinusoidal vessels 15. In their study, HSCs were determined to be relatively distant from both arterioles and transition zone vessels and the location was independent of quiescence 15.

Given that HSCs are mobile and in constant low‐level flux 67, the possibility exists that microscopic images capture a short‐lived interaction of HSCs with neighboring vascular and perivascular niche cells. Using sequential high‐resolution, three‐dimensional imaging of the calvarium of mice over time 68, 69, primitive HSCs have been shown to traffic to BM vessels, where the chemokine CXCL12 and the glycoprotein E‐selectin were rich. HSCs were shown to lodge in these areas for weeks, wherein they underwent self‐renewal and expansion 68, 70.

## BM Osteolineage Cells

The concept of an endosteal niche for HSCs evolved from initial studies that indicated that BM stromal cells could maintain HSCs ex vivo 71, that hematopoietic stem/progenitors localized near endosteal margins 72, and that osteoblasts produce cytokines that support hematopoietic cells 73. Calvi et al. showed that amplification of BM osteoblasts and trabecular bone content led to expansion of the HSC pool in vivo, and Zhang et al. suggested that amplification of N‐cadherin^+^CD45^−^ osteoblastic cells correlated with increased HSC numbers 74, 75. Other studies also suggested that transplanted HSCs resided preferentially in the BM endosteal area in recipient mice 70, 76, 77.

Endochondral ossification, the process of bone formation through a cartilage intermediate, has also been shown to be required for HSC niche formation in mice 78. A clonal, lineage‐restricted common skeletal progenitor cell (bone, cartilage, stromal progenitor) has also been described that contributes CD105^+^, Thy1^+^, and 6C3^+^ stromal cells capable of supporting hematopoietic stem and progenitor cells 79. The maintenance of quiescent HSCs with preserved long‐term repopulating capacity has also been suggested to be dependent on the presence of BM osteolineage cells 80, 81.

Recent studies have challenged whether BM osteoblasts are necessary for HSC maintenance. Several studies have found that most HSCs are not in contact with or in close approximation to BM osteoblasts 59, 65, 68, 82. Second, depleting osteoblasts via *Bgn* deficiency 83 or treatment with ganciclovir 84, 85 had no effect on HSC frequency. Third, increasing BM osteoblast numbers via strontium treatment had no acute effect on HSC frequency 86. It is important to note that the observations of HSC localization and frequency could have been affected in these studies by differences in the criteria used to define the HSC phenotype. However, Ding et al. demonstrated that cell‐specific deletion of stem cell factor (SCF) in BM osteoblasts had no effect on HSC maintenance as measured by competitive repopulation assays in mice 56, 57. Greenbaum et al. also showed that deletion of CXCL12 in BM osteoblasts had no effect on HSC maintenance as measured via competitive repopulation assays 87. Taken together, these data suggest that BM osteoblasts might provide signals that are sufficient to promote HSC expansion; however, it remains unclear whether BM osteolineage cell‐derived signals are necessary for HSC maintenance or regeneration.

## BM ECs in Normal Hematopoiesis and HSC Regeneration

As early as 1961, it was observed that the recovery of hematopoiesis in rats after 10 Gy of total body irradiation (TBI) required the recovery of an intact vasculature 88. In addition, extramedullary hematopoiesis is known to occur in patients in locations devoid of osteolineage cells (e.g., liver and spleen) 89, and ECs were known to create stem cell niches in other tissues, such as the brain 24. Furthermore, given the essential role of ECs in hematopoietic development, investigators have explored the role of ECs in regulating adult hematopoiesis. As noted, HSCs reside in the adult BM in association with vascular and perivascular niche cells 15, 16, 59, 66. In 1972, it was observed that hematopoietic regeneration was linked to vascular regeneration in areas of curetted BM in adult mice 90. More recently, conditional deletion of the gene that encodes the gp130 cytokine receptor in ECs led to a reduction in HSC numbers and overall BM hypocellularity 91.

Ding et al. established the essential role of BM ECs in regulating the maintenance of the HSC pool via cell‐specific deletion of SCF 56. Ding and Morrison 57 and Greenbaum et al. 87 later demonstrated that deletion of *CXCL12* in BM ECs also impaired HSC maintenance in mice. In the same studies, Ding and Morrison demonstrated that deletion of *CXCL12* from LepR^+^ perivascular stromal cells depleted HSCs 57. In contrast, Greenbaum et al. showed that deletion of *CXCL12* from Prx1^+^ mesenchymal progenitor cells markedly decreased HSC content 87. Taken together, these studies confirmed a necessary role for BM ECs and BM perivascular stromal cells in maintaining the HSC pool in steady state.

Human ECs can promote and maintain HSCs in culture 8, 9, 92, and BM ECs promote long‐term reconstituting HSC expansion in culture 2, 93. The BM sinusoidal vasculature is radiosensitive but regenerates and reorganizes within 3–4 weeks after sublethal radiation exposure 10, 94. Following radiation injury, coculture of irradiated HSCs with ECs can rescue HSCs with multilineage reconstituting capacity that are capable of radioprotecting lethally irradiated recipient mice after transplantation 8, 9. Moreover, Chute et al. and Salter et al. demonstrated that systemic infusion of autologous or allogeneic murine ECs into lethally irradiated mice accelerated both BM vascular and hematopoietic regeneration and markedly improved survival, in the absence of transplanted hematopoietic cells 10, 94. Salter et al. demonstrated that transplanted ECs do not engraft in the BM vasculature, suggesting that the regenerative effects were mediated via indirect activities or elaboration of EC‐derived soluble factors 94. This is consistent with clinical studies that have shown that reconstitution of the BM vasculature arises primarily from host BM ECs rather than donor‐derived ECs 95.

Several lines of evidence suggest that BM ECs have a necessary role in HSC regeneration 2, 13, 94. Genetic deletion of *VEGFR2* or antibody‐mediated blockade of VE‐cadherin‐mediated vasculogenesis was shown to disrupt BM vascular regeneration in irradiated mice and result in prolonged hematopoietic toxicity and delayed HSC regeneration 2, 13, 94. Gain of function models have also shown that ECs promote HSC maintenance and regeneration. *Tie2Cre;Bak1^−/−^;Bax^fl/−^* mice, which bear deletion of the intrinsic mediators of apoptosis, BAK and BAX, in Tie2^+^ ECs, were shown to have radioprotection of the BM vasculature and the hematopoietic system compared with mice that retained *BAX* expression in Tie2^+^ ECs 12. Similarly, genetic activation of the Akt‐mTOR pathway in primary human ECs augments the capacity to promote HSC self‐renewal in culture, and mitogen‐activated protein kinase (MAPK) activation favors HSC differentiation in coculture 96. Most recently, the role of SCF‐expressing ECs in promoting extramedullary hematopoiesis in the spleen was also demonstrated in murine models 97. Tcf21^+^ stromal cells were also shown to have a necessary role in maintaining splenic HSCs via CXCL12 secretion in the setting of extramedullary hematopoiesis in the same study 97.

## BM EC‐Derived Paracrine Factors

Although recent studies have confirmed the essential role of BM ECs in maintaining the HSC pool in steady state and regeneration, the precise mechanisms through which BM ECs regulate HSC maintenance and regeneration remain incompletely understood. Although cell‐cell interactions are clearly important, our laboratory has focused on discovering and characterizing the paracrine factors that are produced by BM ECs that regulate HSC fate 92, 98. Some of these factors have been elucidated and well characterized, such as SCF and CXCL12 56, 57, 87. Via an unbiased gene expression analysis of human ECs that supported human HSC expansion 8, Himburg et al. identified pleiotrophin (PTN), a heparin‐binding growth factor, to be more than 25‐fold overexpressed by ECs that promote HSC expansion 18, 99, 100. Ex vivo treatment of murine or human HSCs with PTN caused a marked increase in long‐term repopulating HSCs in culture 18, and systemic administration of PTN to irradiated mice caused a pronounced amplification of BM stem and progenitor cells in vivo 18. The effects were mediated via inhibition of the protein receptor tyrosine phosphatase‐ζ (PTPζ) expressed on HSCs 100. Subsequently, Himburg et al. demonstrated that both BM ECs and CXCL12‐abundant reticular cells express PTN in the HSC niche and that PTN regulates HSC homing to and retention in the BM vascular niche 99. Mechanistically, PTN acts in a paracrine manner in the vascular niche, and binding of PTN with PTPζ on HSCs has been shown to induce phosphorylation of anaplastic lymphoma kinase, yielding downstream activation of Ras/MEK/ERK signaling in the HSC pool 100.

Doan et al. reported that BM ECs in radioprotected *Tie2Cre;Bak1^−/−^;Bax^fl/−^* mice secreted significantly increased concentrations of epidermal growth factor (EGF) and amphiregulin, another EGF receptor (EGFR) agonist, in the BM at steady state and early after myelosuppressive TBI 12. These results suggested that EGF might be produced by Tie2^+^ BM ECs and that EGFR signaling might be functionally relevant to HSC regeneration. Subsequently, Doan et al. showed that systemic administration of EGF promoted HSC regeneration and improved survival in mice after lethal irradiation 101. Mechanistically, EGF promoted HSC regeneration via repression of *PUMA*‐mediated apoptosis in HSCs, leading to increased hematopoietic recovery and improved survival 101.

**Figure 1:**
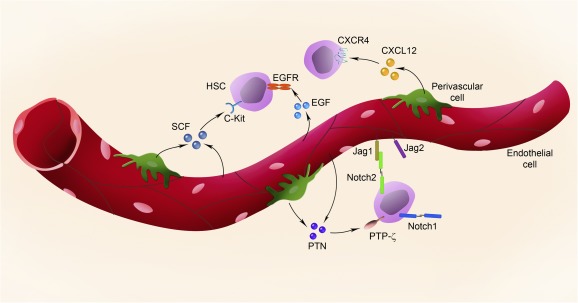
Paracrine factors in the bone marrow (BM) vascular niche. A schematic diagram of a BM vessel in longitudinal view and representation of several paracrine factors that are secreted by BM endothelial cells and perivascular cells. Abbreviations: CXCL12, C‐X‐C chemokine ligand 12; CXCR4, C‐X‐C chemokine receptor type 4; EGF, epidermal growth factor; EGFR, epidermal growth factor receptor; HSC, hematopoietic stem cell; Jag1, Jagged‐1; Jag2, Jagged‐2; PTN, pleiotrophin; PTP‐ζ, protein receptor tyrosine phosphatase‐ζ; SCF, stem cell factor.

Poulos et al. reported that BM ECs also secrete Jagged‐1, a Notch ligand, and conditional deletion of *Jagged‐1* in BM ECs led to hematopoietic exhaustion and HSC depletion over time 17. Hematopoietic regeneration after 650 cGy of TBI was also impaired in mice with EC‐specific Jagged‐1 deficiency 17. Although previous studies have suggested that Notch signaling might be dispensable for normal hematopoiesis 102, the recent studies by Butler et al. and Poulos et al. have indicated that Jagged‐1 signaling, mediated by BM ECs, is necessary for hematopoietic regeneration 2, 17. A schematic representation of the vascular and perivascular‐derived paracrine factors that regulate HSC fate is shown in Figure 1.

## Perivascular Stromal Cells

As noted, several recent studies have highlighted the importance of perivascular cells in regulating HSC fate. Anatomically, BM vessels are ensheathed by pericytes or adventitial reticular cells, including CXCL12‐abundant reticular cells (CAR cells) 55, and other populations characterized by expression of nestin, myxovirus resistance‐1 (Mx1), Lepr, the transcription factor paired related homeobox‐1 (Prx1), and the transcription factor osterix 56, 87, 103, 104. Current understanding indicates substantial overlap in the expression of several of these markers in BM perivascular cells. Pericytes represent structurally unique perivascular cells that express NG2 and surround BM arterioles, whereas LepR^+^ perivascular stromal cells surround BM sinusoidal vessels 16, 105.

The functional role of mesenchymal stromal cells (MSCs) in the HSC niche was suggested by the observation that HSCs reside near nestin‐expressing MSCs and that deletion of nestin‐positive cells depleted BM HSC content 103. Additionally, BM stromal cells with many hallmarks of MSCs express fibroblast activation protein (FAP) 106, 107, and ablation of FAP^+^ cells led to BM hypocellularity and anemia 108, 109. Deletion of the RhoGTPases, Rac1 and Rac2, in nestin‐positive BM cells was associated with decreased nestin‐positive cells, increased trabecular bone, increased sinusoidal space, decreased arteriolar volume, and decreased HSCs in the BM 110. Increased HSC cycling was observed after conditional deletion of NG2‐expressing pericytes, which envelope BM arterioles, suggesting that HSC quiescence is maintained near BM arterioles and is mediated, at least in part, by arteriolar pericytes 16. Ding et al. found that deletion of *SCF* or *CXCL12* from nestin‐positive MSCs had no effect on HSC maintenance 56, 57 but that deletion of *CXCL12* from LepR^+^ perivascular stromal cells caused a significant depletion of HSCs. Greenbaum et al. also demonstrated that deletion of *CXCL12* from nestin‐negative, Prx1‐positive mesenchymal cells yielded HSC depletion 87. Taken together, these studies reveal an essential role for BM perivascular stromal cells in regulating HSC maintenance in vivo. In light of the recent study by Itkin et al., which demonstrated that less permeable arterial blood vessels maintain HSCs in a low ROS state and higher permeability sinusoidal vessels promote hematopoietic stem/progenitor cell activation 66, it will be important to determined which perivascular stromal cells are associated with these two functionally distinct HSC niches.

The production of extracellular matrix proteins by BM stromal cells and the interaction of these molecules with HSCs have also recently come into focus. Deletion of the glycosyltransferase gene, *Ext1*, in Mx1^+^ BM stromal cells was shown to cause a reduction in heparan sulfate production 111. Decreased heparan sulfate levels promoted hematopoietic stem/progenitor cell mobilization and promoted donor hematopoietic cell engraftment in the absence of conditioning 111. In contrast, deletion of protein tyrosine phosphatase *σ* (PTP*σ*), a receptor for heparan sulfate and chondroitin sulfate, was shown to substantially increase the BM repopulating capacity 112. These results suggest the importance of the interaction of the proteoglycans with HSCs in the stromal cell niche and highlight the need for further study into these important mechanisms of HSC regulation.

## Additional Paracrine Mechanisms in the Vascular and Perivascular Niche

In the present review, we focused on the role of BM vascular and perivascular cells in regulating HSC fate. All the important HSC regulatory mechanisms could not be covered comprehensively in the present review, including the Wnt signaling pathway, the Notch pathway and the Tie2/angiopoietin pathway. The function of Wnt ligands in the hematopoietic niche has been previously reviewed in detail 113. Several lines of evidence have suggested that Wnt pathway activation in HSCs and progenitors is under a careful balance among the effects of canonical Wnt ligands, noncanonical Wnt ligands, and Wnt antagonists and that the dosage of Wnt signals can mediate distinct effects on HSC self‐renewal and differentiation 113, 114. Angiopoietin‐1 production by BM osteoblasts was shown by Arai et al. to regulate HSC quiescence via action on Tie2^+^ HSCs 115. More recently, deletion of angiopoietin‐1 from HSCs or LepR^+^ perivascular stromal cells was shown to accelerate hematopoietic and vascular recovery in mice while increasing vascular permeability 116. These data suggest that HSCs and perivascular cells elaborate soluble factors that regulate the vascular response to injury and hematopoietic regeneration.

Although it was not the focus of the present review, recent studies have suggested that corruption of BM niche cells might contribute to or promote the transformation of hematopoietic progenitor cells to malignancy 117, 118, 119. Deletion of the microRNA processing endonuclease, Dicer1, in BM osteoprogenitor cells promoted myelodysplasia in mice 118. In contrast, mutation in β‐catenin in BM osteoblasts promoted acute myeloid leukemia (AML) development in mice 119. It remains unclear whether mutations in BM ECs also contribute to the pathogenesis of myeloid or lymphoid neoplasms, and this is the subject of ongoing study. It has also been shown that AML cells release exosomes containing protein and RNAs that cause downregulation of SCF and CXCL12 in BM MSCs, thereby promoting normal HSC mobilization from the niche and clonal dominance by the AML clone 120, 121. These data suggest that leukemic cells actively participate in subverting the normal HSC niche for the purpose of facilitating AML growth at the expense of normal hematopoiesis.

## Extramedullary Hematopoiesis in Vascular Niches

In the setting of myelofibrosis, chronic myelogenous leukemia, and other myelophthisic processes and stress conditions, extramedullary hematopoiesis can occur in humans in various organs, including the liver, spleen, lymph nodes, retroperitoneum, lungs, genitourinary system, skin, muscle, and rarely, the central nervous system 122, 123. These clinical observations have suggested that these organs must contain an adequate microenvironment to sustain hematopoiesis or that migrating HSCs are capable of modulating the microenvironment in extramedullary sites to support hematopoiesis temporarily. Recently, Inra et al. demonstrated that splenic ECs and Tcf21^+^ stromal cells in the spleen supported EMH via elaboration of SCF or CXCL12 97. Mendt and Cardier also showed that liver sinusoidal ECs “recruit” hematopoietic stem/progenitor cells via secretion of CXCL12 124, and Wittig et al. showed that liver sinusoidal endothelial cells (LSECs) also support B lymphopoiesis 125. These results are consistent with the established role of LSECs in promoting liver regeneration 126 and also are consistent with the recent demonstration that hematopoiesis in the fetal liver occurs in association with fetal portal vessels encompassed by nestin‐positive NG2^+^ pericytes and that such fetal liver hematopoiesis ceases with closure of the umbilical vein and the loss of nestin‐positive NG2^+^ pericytes as the portal vessels transition from the arterial to the venous phenotype 7.

## Opportunities for Translation

Elucidation of the mechanisms through which BM ECs, perivascular cells, and osteolineage cells regulate HSC self‐renewal, regeneration, and malignant transformation will increase the potential for development of targeted therapeutic agents for the human hematopoietic system. Novel vascular niche‐derived paracrine factors that regulate HSC fate are particularly exciting in this regard because the systemic administration of such factors could be readily developed for clinical testing. Several BM EC‐derived soluble proteins described in the present study, such as PTN and EGF, and derivatives of these proteins, are in advanced preclinical testing for investigational new drug evaluation. The ongoing discovery of novel mechanisms through which vascular, perivascular, and osteolineage cells regulate hematopoiesis, coupled with the explication of the cross‐talk signals among BM ECs, perivascular cells, osteolineage cells, and other niche elements, will certainly yield many exciting tools for regenerative medicine.

## Author Contributions

J.P.S., K.T.Y., and J.P.C.: manuscript writing, final approval of manuscript.

## Disclosure of Potential Conflicts of Interest

The authors indicated no potential conflicts of interest.
